# The Norwegian public’s ability to assess treatment claims: results of a cross-sectional study of critical health literacy

**DOI:** 10.12688/f1000research.21902.1

**Published:** 2020-03-11

**Authors:** Astrid Dahlgren, Kjetil Furuseth-Olsen, Christopher James Rose, Andrew David Oxman

**Affiliations:** 1 Centre for Informed Health Choices, Norwegian Institute of Public Health, Postboks 222 Skøyen, Oslo, 0213, Norway

**Keywords:** health literacy, critical health literacy, evidence-informed decision-making, evidence-based practice, critical thinking, public health, shared decision making, public understanding of science

## Abstract

**Background**: Few studies have evaluated the ability of the general public to assess the trustworthiness of claims about the effects of healthcare. For the most part, those studies have used self-reported measures of critical health literacy.

**Methods**: We mailed 4500 invitations to Norwegian adults. Respondents were randomly assigned to one of four online tests that included multiple-choice questions that test understanding of Key Concepts people need to understand to assess healthcare claims. They also included questions about intended behaviours and self-efficacy. One of the four tests was identical to one previously used in two randomised trials of educational interventions in Uganda, facilitating comparisons to Ugandan children, parents, and teachers. We adjusted the results using demographic data to reflect the population.

**Results**: A total of 771 people responded. We estimate that at least half of Norwegian adults understand 18 of the 30 Key Concepts (i.e. the adjusted proportion of correct answers was ≥ 50%). On the other hand, less than half understood 13 of the concepts. The results for Norwegian adults were better than the results for Ugandan children in the intervention arm of the trial and parents, and similar to those of Ugandan teachers in the intervention arm of the trial. Based on self-report, most Norwegians are likely to find out the basis of treatment claims, but few consider it easy to assess whether claims are based on research and to assess the trustworthiness of research.

**Conclusions**: Norwegian adults do not understand many concepts that are essential for assessing healthcare claims and making informed choices. This can result in poorly informed decisions, underuse of effective interventions, and overuse of ineffective or harmful interventions.

## Introduction

Enabling people to make informed decisions about healthcare by improving their ability to think critically about such claims is an important public health initiative ^1^. Health literacy has been defined in many ways. Commonly this includes functional, interactional, and critical health literacy skills ^2^. Health literacy research efforts in Norway and elsewhere have mainly been directed at providing reliable health information and education in functional literacy, focusing on improving understanding of medical terminology and self-management of health conditions ^3^. Very little research has targeted critical health literacy, focused on enabling members of the public to make informed healthcare choices ^4– 6^.

Training in critical thinking about treatment decisions includes courses in evidence-based practice and education in basic research methodology. Such training is usually directed at health professionals. Despite the importance of enabling patients and the public to make informed decisions about treatment choices, such training has rarely been offered ^4– 8^.

Over the past decade, there has been increasing interest in enabling patients and the public to think critically about healthcare ^4, 5, 9– 14^. For example, in Norway three websites have been developed that aim to empower patients and the public to assess the trustworthiness of health claims ^15– 17^. Critical thinking has also been the focus of popular television shows that target common claims about treatment effects and illustrate how such claims can be tested using rigorous study designs. In collaboration with the Norwegian national television network (NRK), we carried out randomised trials with the aim of educating the public about the need for fair comparison of treatments ^18– 22^. More recently, we have sought to improve critical thinking in school children through the Informed Health Choices (IHC) project, an international collaboration of researchers in Uganda, Rwanda, Kenya, the UK, and Norway. We developed an educational intervention to teach primary school children to assess healthcare claims and an educational podcast for their parents. Both interventions were shown to be effective in randomized trials in Uganda ^23, 24^.

Few studies have been done to evaluate this ability in the general public to assess the trustworthiness of claims about the effects of healthcare and to make informed health choices ^6^. Most such studies have relied on self-assessment of critical health literacy skills. One such project is the HLS-EU survey, mapping health literacy skills in several European countries ^25^.

### The Claim Evaluation Tools item bank

The Claim Evaluation Tools item bank was originally developed in English for use in the two randomised trials described above ^23, 24^. Since the item bank was first created items have been translated and validated in several settings and languages, including Uganda (in Luganda and English), Mexico, and China ^26– 30^.

Our starting point for developing the educational interventions and the Claim Evaluation Tools item bank was a list of Key Concepts that people need to understand and apply to assess claims about treatment effects and make informed health choices (hereafter referred to as Key Concepts) ^31^ ( Table 1). We define “treatment” broadly to include any intervention (action) intended to improve health, including preventive, therapeutic and rehabilitative interventions, and public health or health system interventions. The list of concepts provides a framework for researchers, teachers and others to develop interventions and measure people’s ability to assess treatment claims and make informed choices. We review and amend the list yearly ^9^.

**Table 1.** The Informed Health Choices Key Concepts for assessing claims about treatment effects and making well informed treatment choices.

1. Claims *Claims about effects that are not* *supported by evidence from fair* *comparisons are not necessarily wrong,* *but there is an insufficient basis for* *believing them.*	2. Comparisons *Studies should make fair comparisons,* *designed to minimize the risk of* *systematic errors (biases) and random* *errors (the play of chance).*	3. Choices *What to do depends on judgements about* *a problem, the relevance of the evidence* *available, and the balance of expected* *benefits, harms, and costs.*
**1.1 It should not be assumed that** **treatments are safe or effective - or that** **they are not.** a) Treatments can cause harms as well as benefits. b) Large, dramatic effects are rare. c) It is rarely possible to be certain about the effects of treatments. **1.2 Seemingly logical assumptions are not** **a sufficient basis for claims.** a) Treatment may not be needed. b) Beliefs alone about how treatments work are not reliable predictors of the presence or size of effects. c) Assumptions that fair comparisons are not relevant can be misleading. d) An outcome may be associated with a treatment but not caused by it. e) More data is not necessarily better data. f) Identifying effects of treatments depends on making comparisons. g) The results of one study considered in isolation can be misleading. h) Widely used treatments or those that have been used for decades are not necessarily beneficial or safe. i) Treatments that are new or technologically impressive may not be better than available alternatives. j) Increasing the amount of a treatment does not necessarily increase its benefits and may cause harm. k) Earlier detection of ‘disease’ is not necessarily better. l) It is rarely possible to know in advance who will benefit, who will not, and who will be harmed by using a treatment. **1.3 Trust in a source alone is not a** **sufficient basis for believing a claim.** a) Your existing beliefs may be wrong. b) Competing interests may result in misleading claims. c) Personal experiences or anecdotes alone are an unreliable basis for most claims. d) Opinions alone are not a reliable basis for claims. e) Peer review and publication by a journal do not guarantee that comparisons have been fair.	**2.1 Comparisons of treatments should** **be fair.** a) Comparison groups should be as similar as possible. b) Indirect comparisons of treatments across different studies can be misleading. c) The people being compared should be cared for similarly apart from the treatments being studied. d) If possible, people should not know which of the treatments being compared they are receiving. e) Outcomes should be assessed in the same way in all the groups being compared. f) Outcomes should be assessed using methods that have been shown to be reliable. g) It is important to assess outcomes in all (or nearly all) the people or subjects in a study. h) People’s outcomes should be counted in the group to which they were allocated. **2.2 Syntheses of studies need to be** **reliable.** a) Reviews of studies comparing treatments should use systematic methods. b) Failure to consider unpublished results of fair comparisons may result in estimates of effects that are misleading. c) Treatment claims based on models may be sensitive to underlying assumptions. **2.3 Descriptions should clearly reflect** **the size of effects and the risk of being** **misled by the play of chance.** a) Verbal descriptions of the size of effects alone can be misleading. b) Relative effects of treatments alone can be misleading. c) Average differences between treatments can be misleading. d) Small studies may be misleading. e) Results for a selected group of people within a study can be misleading. f) Confidence intervals should be reported for estimates of effects. g) Deeming results to be “statistically significant” or “nonsignificant” can be misleading. h) Lack of evidence of a difference is not the same as evidence of “no difference”.	**3.1 Problems and options should be clear.** a) Be clear about what the problem or goal is and what the options are. **3.2 Evidence should be relevant.** a) Attention should focus on all important effects of treatments, and not surrogate outcomes. b) There should not be important differences between the people in studies and those to whom the evidence will be applied. c) The treatments compared should be similar to those of interest. d) There should not be important differences between the circumstances in which the treatments were compared and those of interest. **3.3 Expected advantages should** **outweigh expected disadvantages.** a) Weigh the benefits and savings against the harms and costs of acting or not. b) Consider the baseline risk or the severity of the symptoms when estimating the size of expected effects. c) Consider how important each advantage and disadvantage is when weighing the pros and cons. d) Consider how certain you can be about each advantage and disadvantage. e) Important uncertainties about the effects of treatments should be reduced by further fair comparisons.

The item bank is an open-access resource. When we conducted this study, it included at least four multiple-choice questions (MCQs) for 32 of the Key Concepts shown in Table 1. The item bank also includes questions that assess intended behaviours and self-efficacy. Teachers and researchers can select items for tests tailored to their specific needs.

To our knowledge only one survey has attempted to measure the ability to understand and apply any of the IHC Key Concepts in a representative sample of Norwegian adults ^32^. That study only addressed four of the Key Concepts. The purpose of this study was to map the ability of Norwegian adults to assess treatment claims and make informed health choices, using MCQs from the Claim Evaluation Tools item bank. The findings can be used to inform the development of learning resources and communication of information to patients and the public, and for international comparisons.

## Objective

To map the ability of Norwegian adults to assess treatment claims and make informed choices, and to compare these results to the findings of two studies in Uganda ^23, 24^.

## Methods

### Ethical statement

This study was considered for ethical approval by the Norwegian Institute of Public Health. This project was considered to not to require a full ethical review (reference 18/11854), because no sensitive data was included. All information was collected through Nettskjema (a web-based survey system), ascertaining a high level of data security and safety.

The population was drawn from the National registry who provided us with a CD-ROM with respondents’ addresses. This CD was destroyed once data collection was terminated.

All respondents were given information about the purpose of the study and how the results would be managed and presented. To gain access to the questionnaire, each participant had to provide written consent electronically. The questionnaire was anonymous and once submitted, the information could not be traced back to the respondent.

### Description of the questionnaires

The Claim Evaluation Tools item bank was developed in English. For this study, a language specialist (KFO) translated all items to Norwegian. Three researchers trained in evidence-based medicine and epidemiology reviewed this translation (AD, ADO, and Atle Fretheim). The questions were not modified other than changing some of the names of people in the scenarios to more familiar names in Norwegian. For example, “Dr. Acheng” was changed to “Dr. Anker” (a more familiar name in Norwegian). MCQs for 32 Key Concepts were administrated as part of this study. However, as previously mentioned, the Key Concepts are reviewed and revised annually. Over the duration of this study, two Key Concepts were revised into the new Key Concept 1.1a. Considering that the MCQs were no longer appropriately covering this concept, these MCQS were therefore taken out of the analysis.

All MCQs in the item bank include a scenario with a treatment claim, and response options, with one answer being the “best” (correct) and the remaining options considered “worse” (incorrect). An example of a multiple-choice question is shown in Box 1. We planned on including questions about intended behaviours and self-efficacy, in addition to two MCQs for each Key Concept. Anticipating that few respondents would be prepared to answer this number of MCQs, we split the questions across four tests (see Additional file 1 and Additional file 5, *Extended data*) ^33, 34^.

**Box 1. Example of a multiple-choice question**

Judith wants smoother skin. The younger girls in her school have smoother skin than the older girls. Judith thinks this is because the younger girls use cream on their skin to make the skin smoother.

*Question:* **Based on this link between using cream and smooth skin, is Judith correct?**

*Options*:

**A**) It is not possible to say. It depends on how many younger and older girls there are
**B**) It is not possible to say. There might be other differences between the younger and older girls
**C**) Yes, because the younger girls use cream on their skin and they have smoother skin
**D**) No, Judith should try using the cream herself to see if it works for her

We included a common set of eight MCQs in all four tests, two for each of four Key Concepts:

- An outcome may be associated with a treatment but not caused by it.
- The results of one study considered in isolation can be misleading.
- Widely used treatments or those that have been used for decades are not necessarily beneficial or safe.
- Comparison groups should be as similar as possible.

We considered these four Key Concepts to be amongst the most important Key Concepts that can be understood and used by children, as well as adults. These MCQs were also included in the test used in the randomised trials in Uganda ^35^.

The first test was identical to the test used for the randomised trials in Uganda ^23, 24^, facilitating comparison between Norwegian adults and Ugandan children, parents, and teachers. The second test included the remaining items that address concepts in the first (Claims) category as well as items from the second (Comparisons) category ( Table 1). The third test included items from the third (Choices) category. This test also included three items evaluating intended behaviours regarding assessing treatment claims and agreeing to participate in a study evaluating treatments, and four items regarding self-efficacy. The fourth test included MCQs addressing Key Concepts that we judged to be more difficult, mostly belonging to the Comparisons category.

All four tests included the same set of questions regarding the participant’s sex, level of education, health professional background, training in research methods, and prior participation in research.

### Recruitment of participants and administration of the questionnaires

**Figure 1.**
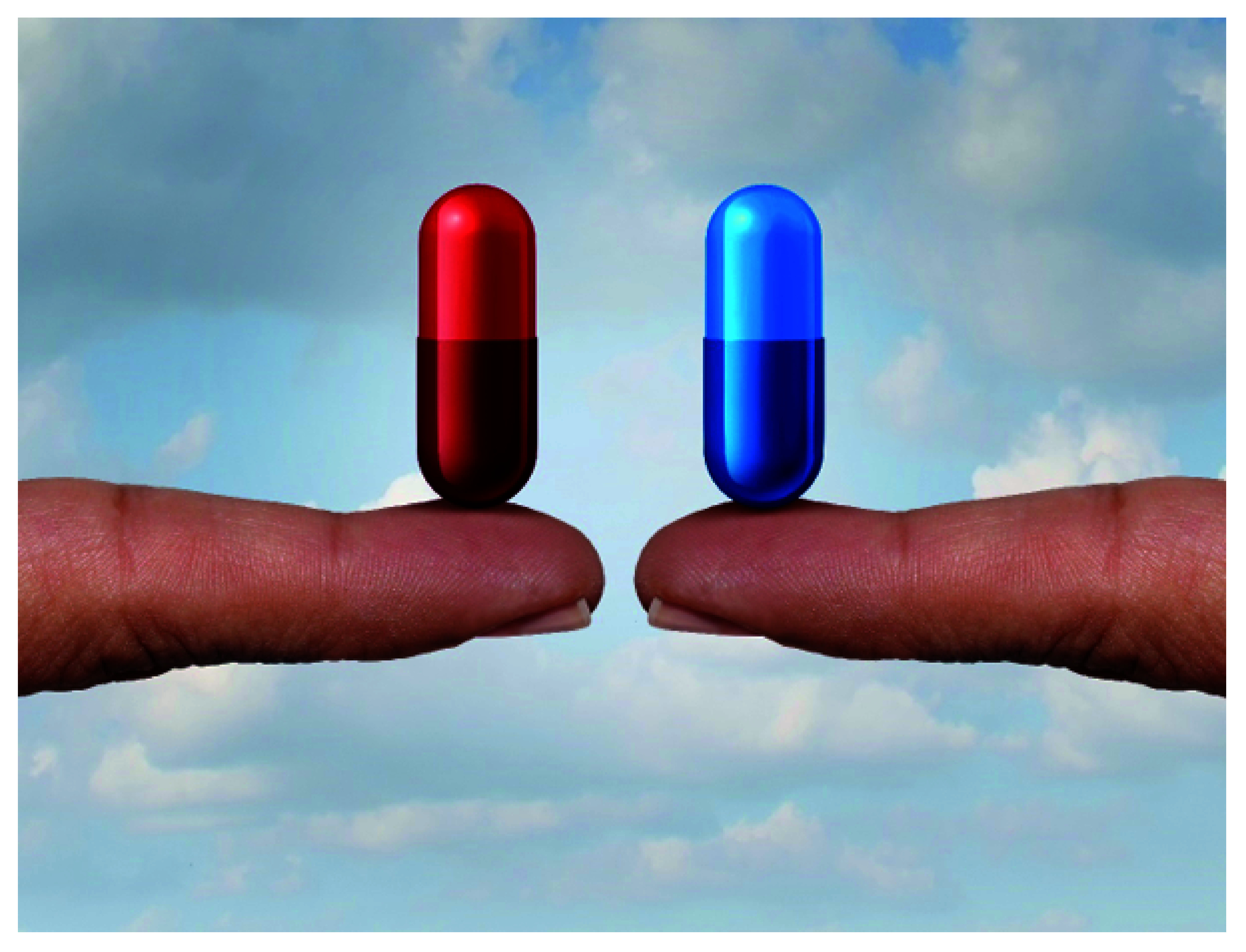
The postcard used for recruitment.

Based on evidence from two systematic reviews that evaluated strategies to improve participation in research ^36, 37^, we developed attractive postcards personally addressed to each potential participant inviting people to take part in the study. The postcard is shown in Figure 1. The postcard included a short description of the purpose of the study as well as a URL (“link”) to one of the four online tests. Participants received full information about the study when they accessed the website and were asked to provide informed consent. Evidence also suggests greater participation rates can be expected if people are informed how the research may benefit them. This was stated on the postcard and the website provided information about how the study results can be accessed.

In January and February 2019, we mailed postcards to a representative sample of 4500 adults (≥18 years) living in Norway. The sample was provided to us by the Norwegian National Registry, considering level of education, sex and geographical spread ^38^. The questionnaires were administrated electronically using Nettskjema, a service provided by University of Oslo ^39^. In addition to the first postcard, one reminder postcard was sent out to each person.

### Rasch analysis

The tests were evaluated for their psychometric properties as part of this study. Although some MCQs were tested in Norway as part of a previous pilot, this is the first full-scale psychometric evaluation of the complete available battery of the Claim Evaluation Tools Item bank ^26, 27^.

Rasch analysis is a dynamic and practical approach to address important measurement issues required for validating an outcome measure and can be used for both dichotomous and polytomous data ^40– 42^. In this study we followed the fundamental steps of Rasch analysis including testing for internal construct validity (multidimensionality), invariance of the items (Item-Person Interaction), and item bias (differential item functioning), as well as testing for reliability ^41, 42^. Rasch analysis can be used for both dichotomous and polytomous data ^41, 43, 44^. Raw data were exported from the electronic data collection service (Nettskjema) as Excel-files and entered into RUMM2030 for Rasch analysis. Results can be replicated using the open-access software WINSTEPS ^45^. Each test set was evaluated separately. Test 3 included also polytomous questions assessing intended behaviour and self-efficacy questions. Therefore, for test 3 specifically, additional analysis was done for each item block separately (MCQ- item block and intended behaviour and self-efficacy questions-block respectively).

### Survey analysis

Each MCQ was scored as correct or incorrect. The Key Concepts for which there were two MCQs were scored as “understood” if a participant answered both MCQs correctly. For questions about intended behaviours and self-efficacy, we dichotomised the responses in the analysis, for example as likely (very likely or likely) vs not likely (very unlikely, unlikely, or don’t know).

Based on a previous survey we conducted ^32^, we anticipated that women and respondents with higher education levels would be more likely to respond. To address such non-random non-response, we used iterative post-stratification to match marginal distributions of sex and educational attainment level of the sample to the Norwegian population ^46^. We also adjusted for region of residence. Due to an error, we did not collect data on participants’ age and could not use it for post-stratification, as planned.

We obtained Eurostat data on the marginal distributions of sex and region of residence for all Norwegians, and educational attainment level for Norwegians aged 15 to 64 years ^47, 48^. Participants reported their county of residence. We mapped counties to the corresponding Nomenclature of Territorial Units for Statistics (NUTS 2) regions of Norway used by Eurostat ^49^. Participants reported the highest level of education they attained. We mapped these to the corresponding International Standard Classification of Education (ISCED) 2011 categories used by Eurostat (levels 0–2, 3–4, and 5–8). We used multiple imputation with chained equations to account for the uncertainty introduced by missing values of the post-stratification variables sex, region of residence, and educational level ^50^, and iteratively post-stratified each imputed data set. Based on these adjustments, we estimated the percentage of the Norwegian population that understands each key concept, and that responded positively for each question about intended behaviours and self-efficacy. We used the R packages tidyverse, mice, mitools, and survey.

We present summaries of the results for participants and the post-stratified population estimates for understanding of the 30 Key Concepts, and the intended behaviours and self-efficacy questions. We quantified the uncertainty of our estimates using 95% confidence intervals and protected the family-wise coverage probability of the confidence intervals for the four Key Concepts included in all questionnaires via Bonferroni-correction (i.e., we report a 98.75% CI for each of those concepts). For each Key Concept, we calculated the likelihood of answering both questions (or the one question for two Key Concepts) correctly if participants randomly guessed the answer. These probabilities vary between six and 25%, depending on the number of MCQs and the number of response options (between two and four) for each MCQ.

We compared Norwegian and Ugandan adults’ understanding of the Key Concepts to that of Ugandan children in the intervention arm of our randomized trial. We compared mean test scores, and probabilities of achieving passing and mastery scores, using estimates from one year after the IHC primary school intervention for the Ugandan children and their teachers ^51, 52^ and estimates from the control group for parents in the IHC podcast trial, one year after parents in the intervention group listened to the podcast ^52, 53^. We used predetermined thresholds of at least 13 of 24 questions answered correctly for a passing score and at least 20 of 24 for mastery.

We estimated mean scores and odds ratios using generalized linear mixed-effects models (GLMMs; normal errors and identity link for mean scores, binomial errors and logit link for passing and mastery) using the lme4 R package. In the trials, children and teachers were randomized in clusters (schools), while parents were individually randomized. We modelled this clustering structure as a random intercept for each randomized unit. We did not adjust for covariates such as those used for stratified sampling in the Ugandan trials because those variables are not defined for all samples (e.g., school ownership was used in the trial on Ugandan children, but there is no analogous concept for Norwegian adults). No data were missing. It is not possible to use the lme4 package to apply post-stratification weights for the Norwegians.

### Exploratory analyses

We conducted exploratory analyses to investigate associations between understanding each Key Concept and the demographic covariates sex, research training, research participation, education level, and health professional background. Based on findings from our previous survey ^32^, we hypothesised that better understanding of the Key Concepts would be associated with having a research background or a higher education level; that there would be little difference between health professionals and others; and that there would be little difference between women and men. We used generalized linear models (GLMs; quasibinomial errors and logit link) as before, modelled the covariates as categorical variables, and used multiple imputation and post-stratification as in the main analysis.

We used data from the first questionnaire to perform exploratory analyses to investigate how Norwegians’ mean scores and achievement of passing and mastery scores are associated with the demographic covariates sex, research training, research participation, education, and health professional background. We used GLMs (normal errors and identity link for mean score; quasibinomial errors and logit link for passing and mastery) to model the outcomes in terms of the covariates, which were modelled as before. Multiple imputation and post-stratification were used as in the main analysis. The variables included in imputation were the post-stratification variables (sex, region of residence, and educational attainment); demographic variables that coded for whether participants had research training or a health professional background, and whether they had been a research participant; and mean score. We did not include passing and mastery in imputation, as they can be calculated from the mean score.

### Sample size calculation

We performed three power analyses. The first two analysed power to estimate differences, of at least 5% from random guessing, in the proportion of the Norwegian population that understands the concepts. The first analysis focused on the four Key Concepts that would be included on all four tests while protecting the 95% coverage of the four confidence intervals as a family, and the second analysis focused on a single prototypical Key Concept that would be included on only one test. We assumed that each concept would be probed by two MCQs, each having four options, such that we would expect that 6.25% of Norwegians would appear to “understand” a concept if all participants guessed at random. We further calculated confidence interval widths, aiming to estimate proportions with precision no worse than ±0.05 (i.e., ±5%) for those four Key Concepts and no worse than ±0.08 (i.e., ±8%) for the other Key Concepts. The third analysis estimated power to detect a difference of at least 10% in the mean score (proportion of correct answers) between Norwegians and Ugandan children, parents, and teachers. Finally, drawing on experience with our previous survey, we explored a range of response rate scenarios.

These analyses suggested that sending a total of 4500 postcards would be sufficient to achieve a margin of error no greater than ±5% and ±8% for the first two analyses, and approximately 97% power to detect differences greater than 10% in the mean score compared to results from the Ugandan trials.

## Results

### Participant characteristics

Table 2 shows participant characteristics across the four questionnaires ^54^. Of the 771 respondents, 21 (2.7%) did not provide data on at least one of the covariates sex, research training, research participation, education, health professional background, and county of residence.

**Table 2.** Participant characteristics across the four questionnaires.

		Quiz 1	Quiz 2	Quiz 3	Quiz 4	P-value	Overall
All		210 (27.2%)	211 (27.4%)	172 (22.3%)	178 (23.1%)	1	771 (100.0%)
Sex	Female	103 (13.4%)	102 (13.2%)	102 (13.2%)	94 (12.2%)	0.63355	401 (52.0%)
	Male	107 (13.9%)	109 (14.1%)	70 (9.1%)	83 (10.8%)	0.56449	369 (47.9%)
	Missing	0 (0.0%)	0 (0.0%)	0 (0.0%)	1 (0.1%)	0.45466	1 (0.1%)
Education	Primary	12 (1.6%)	16 (2.1%)	5 (0.6%)	10 (1.3%)	0.30396	43 (5.6%)
	Secondary	53 (6.9%)	53 (6.9%)	42 (5.4%)	49 (6.4%)	0.95886	197 (25.6%)
	Tertiary (1–2 years)	23 (3.0%)	30 (3.9%)	21 (2.7%)	33 (4.3%)	0.29255	107 (13.9%)
	Tertiary (3–5 years)	71 (9.2%)	69 (8.9%)	58 (7.5%)	51 (6.6%)	0.86021	249 (32.3%)
	Masters degree	39 (5.1%)	36 (4.7%)	41 (5.3%)	30 (3.9%)	0.49704	146 (18.9%)
	PhD	10 (1.3%)	5 (0.6%)	5 (0.6%)	3 (0.4%)	0.38534	23 (3.0%)
	Missing	2 (0.3%)	2 (0.3%)	0 (0.0%)	2 (0.3%)	0.67033	6 (0.8%)
Research training	No	126 (16.3%)	145 (18.8%)	105 (13.6%)	126 (16.3%)	0.66839	502 (65.1%)
	Yes	84 (10.9%)	66 (8.6%)	65 (8.4%)	52 (6.7%)	0.35023	267 (34.6%)
	Missing	0 (0.0%)	0 (0.0%)	2 (0.3%)	0 (0.0%)	0.050443	2 (0.3%)
Research participant	No	146 (18.9%)	157 (20.4%)	121 (15.7%)	126 (16.3%)	0.97134	550 (71.3%)
	Yes	63 (8.2%)	52 (6.7%)	50 (6.5%)	51 (6.6%)	0.80132	216 (28.0%)
	Missing	1 (0.1%)	2 (0.3%)	1 (0.1%)	1 (0.1%)	1	5 (0.6%)
Medical education	No	175 (22.7%)	168 (21.8%)	133 (17.3%)	147 (19.1%)	0.96116	623 (80.8%)
	Yes	35 (4.5%)	43 (5.6%)	37 (4.8%)	28 (3.6%)	0.57834	143 (18.5%)
	Missing	0 (0.0%)	0 (0.0%)	2 (0.3%)	3 (0.4%)	0.061528	5 (0.6%)
County	Akershus	27 (3.5%)	28 (3.6%)	16 (2.1%)	21 (2.7%)	0.72691	92 (11.9%)
	Aust-Agder	4 (0.5%)	2 (0.3%)	2 (0.3%)	2 (0.3%)	0.87829	10 (1.3%)
	Buskerud	7 (0.9%)	7 (0.9%)	14 (1.8%)	8 (1.0%)	0.162	36 (4.7%)
	Finnmark	5 (0.6%)	2 (0.3%)	3 (0.4%)	1 (0.1%)	0.48594	11 (1.4%)
	Hedmark	2 (0.3%)	3 (0.4%)	4 (0.5%)	3 (0.4%)	0.76607	12 (1.6%)
	Hordaland	16 (2.1%)	29 (3.8%)	9 (1.2%)	20 (2.6%)	0.048787	74 (9.6%)
	Møre og Romsdal	8 (1.0%)	3 (0.4%)	6 (0.8%)	9 (1.2%)	0.24526	26 (3.4%)
	Nordland	9 (1.2%)	9 (1.2%)	5 (0.6%)	6 (0.8%)	0.89401	29 (3.8%)
	Oppland	3 (0.4%)	9 (1.2%)	5 (0.6%)	5 (0.6%)	0.40473	22 (2.9%)
	Oslo	38 (4.9%)	29 (3.8%)	38 (4.9%)	26 (3.4%)	0.27531	131 (17.0%)
	Østfold	11 (1.4%)	9 (1.2%)	14 (1.8%)	7 (0.9%)	0.37221	41 (5.3%)
	Rogaland	25 (3.2%)	35 (4.5%)	18 (2.3%)	20 (2.6%)	0.40053	98 (12.7%)
	Sogn og Fjordane	3 (0.4%)	2 (0.3%)	4 (0.5%)	1 (0.1%)	0.53125	10 (1.3%)
	Telemark	7 (0.9%)	5 (0.6%)	4 (0.5%)	9 (1.2%)	0.47175	25 (3.2%)
	Troms	6 (0.8%)	8 (1.0%)	3 (0.4%)	7 (0.9%)	0.64403	24 (3.1%)
	Trøndelag	19 (2.5%)	14 (1.8%)	13 (1.7%)	15 (1.9%)	0.84835	61 (7.9%)
	Vest-Agder	6 (0.8%)	10 (1.3%)	6 (0.8%)	11 (1.4%)	0.4576	33 (4.3%)
	Vestfold	12 (1.6%)	6 (0.8%)	7 (0.9%)	6 (0.8%)	0.53705	31 (4.0%)
	Missing	2 (0.3%)	1 (0.1%)	1 (0.1%)	1 (0.1%)	1	5 (0.6%)

### The validity and reliability of the questionnaires

The log describing the Rasch analysis is described in more detail in Additional file 2 ( *Extended data*) ^55^. Overall, the four tests showed acceptable fit to the Rasch model. Three out of four tests were found to be unidimensional. The test including intended behaviour and self-efficacy items suggested that this sub-scale may include more than one dimension. Separate analyses of the two item-sets in Test 3 (Key Concept MCQs and intended behaviour and self-efficacy items) suggest that the MCQ sub-test works very well, with no apparent validity issues. Overall, the analysis of the sub-test consisting of items evaluating intended behaviour and self-efficacy items also shows satisfactory fit. However, two of the intended behaviour items show disordered thresholds. We did not observe any important local dependency in Test 2, 3 and 4. However, in Test 1, twelve correlations was observed. Two tests were underpowered for this analysis (Test 1 and 4). However, based on the available data, most MCQs in all tests showed acceptable fit to the ICC-curve, and no important differential item functioning by gender was identified in any of the tests. Five MCQs showed evidence of under discriminating with one ability class deviating: two MCQs in Test 1 (q11 and q19.1), two MCQs in Test 2 (q3 and q18.3), and one MCQ in Test 4. In the two tests that were adequately powered, Cronbach’s alpha was 0.6 and 0.7, respectively.

### Norwegians’ understanding of the Key Concepts

The mean width of the confidence intervals for the four concepts common to all questionnaires was ±4.6% (range ±3.6% to ±5.9%). One concept (An outcome may be associated with a treatment but not caused by it) was not estimated to the desired precision. The mean width of the other confidence intervals was ±7.92% (range ±3.37% to ±11.9%; 15 of the 30 concepts had confidence intervals wider than ±8%).

**Figure 2.**
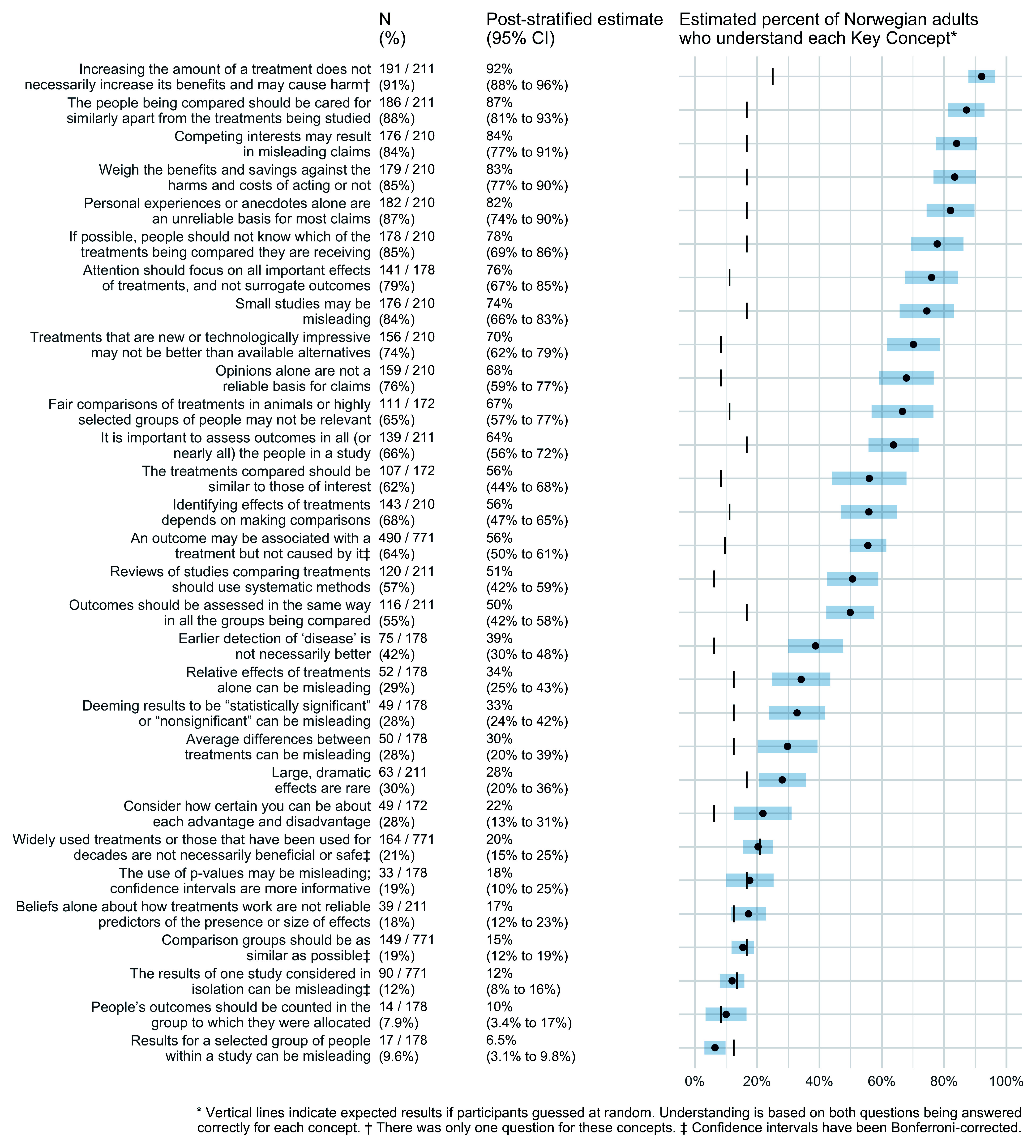
Estimates of the percentage of Norwegian adults who understand each Key Concept.

Figure 2 shows estimates of the percentage of Norwegian adults who understand each Key Concept. We estimate that more than 80% of Norwegian adults understand five of the Key Concepts and more than 50% understand 18 concepts. On the other hand, less than half of Norwegian adults understand 13 of the Key Concepts, and for seven of those concepts, the proportions of correct answers were no better than if people had randomly guessed.

### Intended behaviours and self-efficacy

**Figure 3.**
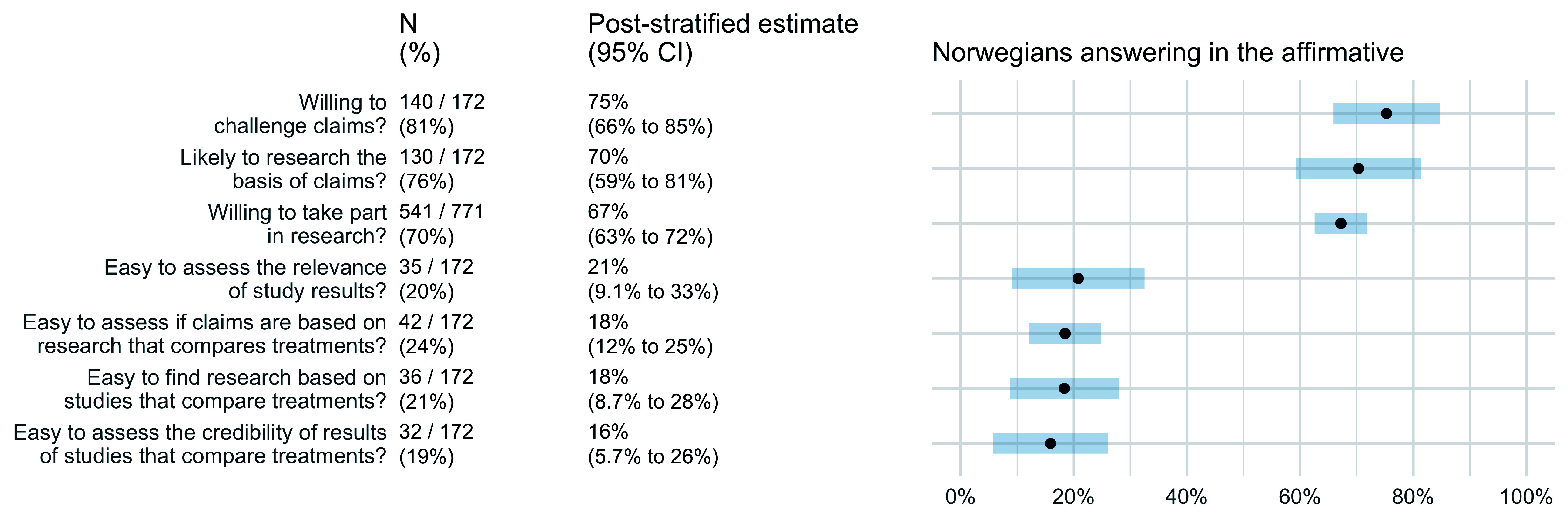
The Norwegian population’s intended behaviours and self-efficacy.

Intended behaviours and self-efficacy are presented in Figure 3. The results suggest that most Norwegian adults are likely to question the basis of treatment claims (75%; 95% CI 66–85%), find out if treatment claims are based on research evidence (70%; 95% CI 59–81%), and say “yes” if asked to participate in a study comparing treatments (67%; 95% CI 63–72%). However, only about one out of five Norwegian adults find it easy to assess the relevance of studies that compare treatments (21%; 95% CI 9–33%), assess whether a treatment claim is based on a study comparing treatments (18%; 95% CI 12–25%), find studies that compare treatments (18%; 95% CI 9–28%), or assess the trustworthiness of the results of studies that compare treatments (16%; 95% CI 6–26%).

### Comparison of Norwegian and Ugandan adults to Ugandan children

The results for Norwegian and Ugandan adults are compared to results for Ugandan children in Table 3 to Table 5. The mean score for Norwegian adults who participated in the survey (Test 1) was 17% higher (95% CI 14–20%) than the mean score for Ugandan children in the intervention arm of the randomised trial of the IHC primary school resources one year after the intervention and similar to the mean score for Ugandan teachers in the intervention arm of the trial ( Table 3) ^23^. The scores for Ugandan teachers in the control group were similar to those of the children in the intervention group, and the scores of Ugandan parents in the control group of the IHC podcast trial ^24^ were 16% lower (95% CI -19 to -13%).

**Table 3.** The mean test scores of the Norwegian and Ugandan samples.

	n	Mean score (95% CI)	Difference (95% CI)	P-value
Ugandan Children (Intervention)	3943	69% (67% to 71%)	0	
Ugandan Parents (Control)	256	53% (48% to 57%)	-16% (-19% to -13%)	<0.0001
Ugandan Teachers (Control)	59	68% (61% to 75%)	-0.59% (-5.6% to 4.4%)	0.82
Ugandan Teachers (Podcast)	78	86% (81% to 92%)	17% (14% to 21%)	<0.0001
Norwegian Adults	210	86% (81% to 91%)	17% (14% to 20%)	<0.0001

**Table 4.** The compared probability of passing in the Norwegian and Ugandan samples.

	Sample		Probability (95% CI)	Difference (95% CI)	Odds ratio (95% CI	P value
Ugandan Children (Intervention)	3160 / 3943	(80.1%)	84% (81% to 88%)	0	1	
Ugandan Parents (Control)	101 / 256	(39.5%)	37% (28% to 47%)	-47% (-56% to -37%)	0.11 (0.073 to 0.17)	<0.0001
Ugandan Teachers (Control)	50 / 59	(84.7%)	88% (76% to 94%)	3.4% (-8.5% to 9.9%)	1.3 (0.58 to 3)	0.4984
Ugandan Teachers (Podcast)	77 / 78	(98.7%)	99% (94% to 100%)	15% (9.7% to 15%)	21 (3 to 150)	0.0023
Norwegian Adults	209 / 210	(99.5%)	100% (98% to 100%)	15% (13% to 16%)	54 (7.5 to 400)	<0.0001

**Table 5.** The compared probability of mastery in the Norwegians and Ugandan samples

	Sample		Probability (95% CI)	Difference (95% CI)	Odds ratio (95% CI)	P value
Ugandan Children (Intervention)	1138 / 3943	(28.9%)	27% (22% to 32%)	0	1	
Ugandan Parents (Control)	27 / 256	(10.5%)	8.1% (5.1% to 13%)	-18% (-22% to -14%)	0.24 (0.15 to 0.4)	<0.0001
Ugandan Teachers (Control)	13 / 59	(22.0%)	19% (9.8% to 33%)	-8% (-17% to 5.9%)	0.63 (0.3 to 1.3)	0.23
Ugandan Teachers (Podcast)	53 / 78	(67.9%)	70% (59% to 80%)	44% (32% to 53%)	6.6 (3.9 to 11)	<0.0001
Norwegian Adults	133 / 210	(63.3%)	66% (56% to 74%)	39% (29% to 48%)	5.3 (3.5 to 7.9)	<0.0001

Nearly all the Norwegian adults (209 out of 210) who participated in the survey and Ugandan teachers in the intervention group (77 out of 78) had a passing score ( Table 4). This was 15% more than the Ugandan children in the intervention group. However, because only one Norwegian and one Ugandan teacher in the intervention group did not have a passing score, the estimated differences may be unreliable for those comparisons. The proportion of Ugandan teachers in the control group with a passing score (88%) was similar to that of the children (84%), and 47% fewer (95% CI -56% to -37%).

Three quarters (66%; 95% CI 56–74%) of the Norwegian adults who participated in the survey and 70% (95% CI 59–80%) of the Ugandan teachers in the intervention group had a mastery score ( Table 5). Compared to Ugandan children in the intervention group, the Odds for achieving a mastery score by the Norwegian participants was 5.3 (95% CI 3.5–7.9%), whereas the Odds for achieving a mastery score was 0.24 (95% CI 0.15–0.4) for the Ugandan parents compared to the children.

### Associations between participant characteristics and their responses

We did not find strong associations between gender, health professional background, or having participated in research and how well participants did ( Table 6). Having 1–2 years of tertiary school education (ISCED levels 5–8) was associated with a mean score that was 11% higher (95% CI 4.0–19%), and a higher likelihood of having a mastery score (OR 3.5; 95% CI 1 to 12). Norwegian men were more likely than women to state that they are likely to find out the basis of treatment claims (OR 3.0; 95% CI 1.1 to 8.2), and more likely to find it easy to assess the trustworthiness of the results of studies that compare treatments (OR 3.8; 95% CI 1 to 14). People who have participated in research may be more likely to find it easy to find research based on studies that compare treatments (OR 5.7; 95% CI 1.9 to 17). People with secondary school education (ISCED levels 3–4) may be less likely than people with no more than primary school education (ISCED levels 0-2) to find it easy to assess the trustworthiness of results of studies that compare treatments (OR 0.057; 95% CI 0.0049 to 0.68). Besides these associations, we did not find evidence suggesting that gender, education level, health professional background, or prior participation in research were associated with intended behaviours or self-efficacy. Associations between participant characteristics and their understanding of specific Key Concepts, intended behaviour and self-efficacy are reported in Additional file 3 ( *Extended data*) ^56^.

**Table 6.** Associations between demographic covariates and Norwegians’ mean scores and achievement of passing and mastery scores

	Intercept	Male	Research participant	ISCED Levels 3-4	ISCED Levels 5–8	Medical education
Mean score	75.0% (68.0% to 82.0%)	-2.3% (-6.6% to 2.1%)	0.6% (-5.5% to 6.7%)	5.0% (-2.9% to 13.0%)	11.0% (4.0% to 19.0%)	-0.4% (-5.2% to 4.4%)
Passing	5.8e+33 (6.9e+32 to 4.9e+34)	6.7e-23 (6.4e-24 to 7.1e-22)	3.8e-23 (5.9e-24 to 2.4e-22)	2e+22 (2.2e+21 to 1.8e+23)	2e+22 (3e+21 to 1.3e+23)	0.28 (0.063 to 1.2)
Mastery	0.53 (0.17 to 1.7)	0.81 (0.36 to 1.8)	1.7 (0.54 to 5.1)	0.89 (0.26 to 3.1)	3.5 (1 to 12)	1.3 (0.42 to 4.1)

ISCED, International Standard Classification of Education.

## Discussion

According to Statistics Norway, 24.7% of Norwegian adults have a primary school education, 41.7% secondary school education, 22.4% tertiary school education, 7.3% have a master’s degree, and 0.7% of the population have a PhD. As we anticipated, participants in our study had a somewhat higher educational level than the general population ^57^. In Norway, approximately 18% of the population (15–74) have an educational background in health or welfare ^58^. This is comparable to participants in this study.

Participants in our survey had a good understanding of the 12 Key Concepts that were addressed in our randomised trial of the IHC primary school intervention ^23^. Their understanding was comparable to Ugandan teachers in the intervention arm of our randomized trial of a primary school intervention, and better than that of Ugandan children in the intervention arm of that trial, and teachers in the control group. It was also better than parents in the control group of our randomised trial of an educational podcast for parents of primary school children in Uganda ^24^.

We estimate that more than 80% of Norwegian adults understand these five concepts:

- Increasing the amount of a treatment does not necessarily increase its benefits and may cause harm.
- Competing interests may result in misleading claims.
- Personal experiences or anecdotes alone are an unreliable basis for most claims.
- The people being compared should be cared for similarly apart from the treatments being studied.
- Weigh the benefits and savings against the harms and costs of acting or not.

On the other hand, Norwegian adults appear to do no better than if they were to randomly guess the answers to questions about these seven Key Concepts:

- Beliefs alone about how treatments work are not reliable predictors of the presence or size of effects.
- Widely used treatments or those that have been used for decades are not necessarily beneficial or safe.
- Comparison groups should be as similar as possible.
- People’s outcomes should be counted in the group to which they were allocated.
- Results for a selected group of people within a study can be misleading.
- Deeming results to be “statistically significant” or “nonsignificant” can be misleading.

Considering that people who responded to our survey had a somewhat higher educational level than the general population, the ability of our respondents may be higher than in the general population ^57^.

Based on self-report, most Norwegians are likely to find out what the basis of a treatment claim is and to find out if a treatment claim is based on research. However, they do not consider it easy to assess the relevance of a claim, whether it is based on research, or its trustworthiness. They also do not consider it is easy to find relevant studies.

### Comparison with other studies

In the previous Norwegian study, four Key Concepts were evaluated ^32^. In both studies the majority responded correctly to the question “Weighing the benefits and harms”. However, in the present study, the respondents were less likely to respond correctly to “Relative effects of treatments can be misleading” (29% vs 64%) and “The use of p-values may be misleading” (19% vs 51.5%). In contrast, more responded correctly to “An outcome may be associate with a treatment but not caused by it” (64% vs 30%). It is difficult to interpret these differences, other than that these evaluations were done using different question sets. Consequently, one explanation may be difference in the questions’ difficulty level. The gender distribution in the studies were identical, however a higher percentage in the present study had at least one year of education beyond secondary school (68% vs 52%). We did not find gender or having a health professional background to be a good predictor of participants’ understanding of the Key Concepts in any of studies. This is consistent with other studies and findings that both health professionals and patients feel challenged finding, appraising, and applying relevant evidence for use in health decisions ^59– 64^.

There are few other studies to which we can compare these results ^6^. The European health literacy survey included all domains of health literacy, responses were given as self-assessments, and results are reported as overall scores ^25^. Consequently, comparison with our study is difficult. They concluded that a little more than one tenth (12%) of those surveyed had insufficient health literacy and almost one half (47%) had limited (insufficient or problematic) health literacy. Both our survey and the European health literacy survey examined associations with education and, unsurprisingly, found that people with higher education have higher health literacy skills. However, the European health literacy survey reported that this result varies by country.

### Strengths and limitations

The strength of this study is that we provide new evidence on people’s ability to assess treatment claims using multiple-choice questions. To our knowledge, few such objective surveys have been conducted in Europe. Our work complements studies in which participants assessed their own abilities.

Many conceptualisations of critical thinking exist. Not all are based on explicit criteria, and unlike the Key Concepts used in this survey, few if any are subject to annual revision ^65^. The questionnaires we administrated were based on a framework that has been developed from methodological literature and input from multiple disciplines and people with methodological expertise ^66^.

We validated the questionnaires using robust methods. Although the results of the Rasch analysis are promising (Additional file 2, *Extended data*) ^55^, it suggests the potential for improvements. Across all four tests, we found that only five MCQs warranted improvement. However, Rasch analysis of two of the tests was underpowered, so the validity and reliability of these should be assessed again in future studies.

Our analysis of the test including intended behaviours and self-efficacy suggests that intended behaviour and self-efficacy items measure two different dimensions. This might be because intended behaviour is more complex and dependent on self-efficacy, values, knowledge, and other factors. Except for Test 1, we did not identify any important dependencies between items.

We did not collect information on age for this study, and thus the association between age and ability to assess treatment claims should be explored in future studies. In our previous study conducted in Norway, younger respondents had a higher proportion of correct responses and higher total scores. This may suggest that there may have been improvements over time in the ability of both health professionals and patients to assess treatment claims ^32^.

Another possible limitation of our study is the low response rate, which we anticipated. Our sample was similar to the general population in terms of the percentage of health professionals, but people with a higher education were over-represented among respondents. We addressed this issue using prespecified post-stratification in the survey analyses but did not address this in the comparisons to Ugandans. The results of those analyses therefore cannot be assumed to apply to the general population of Norwegian adults.

### Implications and future research

The results of this study can inform the development and evaluation of educational interventions that address Key Concepts that Norwegians appear to poorly understand. Up to now, few such interventions have been evaluated. There is a need to evaluate interventions for health professionals as well as for the general public to help ensure that they can think critically about treatment claims and choices. The results also can inform the development and evaluation or strategies for improving communication of information about the effects of treatments by researchers, health professionals, and others.

Studies like this one in other countries would help to map similarities and differences in people’s abilities across different countries and settings. Such information could help to determine the extent to which interventions should be tailored to address different Key Concepts for different populations.

Health professionals and others who communicate health information should be aware that patients may not be able to think critically about treatment claims and may therefore struggle to process information necessary to making informed decisions.

Our Rasch analysis suggests that the MCQs can be used in Norway. Most MCQs performed well, and this evaluation is the first step in developing a calibrated item bank that can be used for Computer Assisted Testing. The results of our Rasch analysis can be used to improve the multiple-choice questions that did not perform well.

## Conclusion

Norwegian adults’ understanding of Key Concepts for assessing treatment claims and making choices varies from over 80% who understand five Key Concepts to 20% or less who understand seven Key Concepts. We did not find strong evidence that gender, being a health professional, or having participated in research are associated with the ability to assess treatment claims, intended behaviours, or self-efficacy. We did find evidence that people with higher education have a better understanding of Key Concepts.

Understanding the need for systematic reviews of fair comparisons as the basis for trustworthy treatment claims has the potential to reduce waste and harm from trusting and acting on misleading claims, and from not trusting and acting on reliable claims. Future interventions should be tailored to address these essential Key Concepts as well as other Key Concepts for which there appears to be a lack of understanding.

## Data availability

### Underlying data

Harvard Dataverse: Replication Data for: Raw data Norwegian Claim Study_2019. https://doi.org/10.7910/DVN/R3DHA5 ^54^

This project contains the following underlying data:

- Quiz-1-data.xlsx
- Quiz-2-data.xlsx
- Quiz-3-data.xlsx
- Quiz-4-data.xlsx

With respect to the potential ability to triangulate identities, access to data is restricted. Ethical approval was granted with an informed consent including a statement were people were guaranteed to be anonymous. Prospective data users can contact the authors to access the data, which can be rejected or approved by the data owner based on certain conditions (must be for legitimate research purposes and requestors are required to sign a data use agreement).

Zenodo: multinormal/fhi.informed-health-choices-norway.2019: Version 1.0. https://doi.org/10.5281/zenodo.3669964 ^52^

This project contains the following underlying data:

- Uganda-children-one-year-follow-up.csv
- Uganda-parents-immediate.csv
- Uganda-parents-one-year-follow-up.csv
- Uganda-teachers-one-year-follow-up.csv

### Extended data

Figshare: Additional file 1. Claim Evaluation Tools_tests administrated in Norway_2019. https://doi.org/10.6084/m9.figshare.11439651.v1 ^33^

Figshare: Additional file 2 Log Rasch analysis Norwegian Claim Study_2019.docx. https://doi.org/10.6084/m9.figshare.10109840.v1 ^55^

Figshare: Additional file 3. Supplementary tables and figures_Norwegian Claim Study_2019. https://doi.org/10.6084/m9.figshare.11439915.v1 ^56^

Figshare: Additional file 5. English versions of Claim Evaluation Tools_tests administrated in Norway_2019. https://doi.org/10.6084/m9.figshare.11652342.v1 ^34^

### Reporting guidelines

Figshare: Additional file 4. STROBE_checklist_cross-sectional_Norwegian Claim Study_2019. https://doi.org/10.6084/m9.figshare.11439753.v2 ^67^

Data are available under the terms of the Creative Commons Attribution 4.0 International license (CC-BY 4.0).

## Software availability

Source code available from: https://github.com/multinormal/fhi.ihc.norway.2019

Archived source code at time of publication: https://doi.org/10.5281/zenodo.3606259 ^52^

License: MIT
