# Supplementary material for: The Norwegian public’s ability to assess treatment claims: results of a cross-sectional study of critical health literacy
Source: F1000Res. 2021 Jul 30;9:179. Originally published 2020 Mar 11. [Version 2] doi: 10.12688/f1000research.21902.2 (PMC10995534; doi:10.12688/f1000research.21902.2)
Supplement: Peer review history

# Reviewer report: Reviewer response for version 2

**DOI:** 10.5256/f1000research.58717.r255278

**Published:** 2024-04-04

**Authors:** Susie Sykes

**Affiliations:** 1 Institute of Health and Social Care, London South Bank University, London, UK

Many thanks for the opportunity to review this paper. It is a very interesting paper and explores an important area of enquiry.

 The paper sets itself up as a cross-sectional study of critical health literacy when in fact it is a cross-sectional study of critical appraisal skills. The two are different and the conceptual basis of the study therefore needs clarification. The literature offering conceptual clarity on critical health literacy has not been drawn upon and therefore key components of the concept including the important element of social and political action are overlooked. The paper should either more accurately reflect critical health literacy as a concept or refer instead to critical thinking or critical appraisal skills.

 The authors should consider literature such as:[1],[2],[3],[4],[5].

 The authors are correct that little has been done to measure CHL but do not refer to work other than the HLS_EU. They should consider:

 [6],[7],[8]

 The last of these was conducted in Norway and would seem extremely relevant.

 The purpose of comparing the Norwegian and Ugandan populations is unclear to me. The rationale for such a comparison is not clearly made. No reference to the comparison is made in the discussion or conclusion so the value of the comparison within the results section is not clear.

 The different context between the two countries is also not properly explored. A statement is given that: it is our experience from previous projects that the challenges associated with assessing claims in everyday life in Uganda and Norway are very similar but this is insufficient.

Is the work clearly and accurately presented and does it cite the current literature?

No

If applicable, is the statistical analysis and its interpretation appropriate?

I cannot comment. A qualified statistician is required.

Are all the source data underlying the results available to ensure full reproducibility?

Yes

Is the study design appropriate and is the work technically sound?

Yes

Are the conclusions drawn adequately supported by the results?

Yes

Are sufficient details of methods and analysis provided to allow replication by others?

Yes

Reviewer Expertise:

public health and health promotion

I confirm that I have read this submission and believe that I have an appropriate level of expertise to confirm that it is of an acceptable scientific standard, however I have significant reservations, as outlined above.

## References

[1] : Understanding and Responding to Health Literacy as a Social Determinant of Health. *Annu Rev Public Health* .2021;42: 10.1146/annurev-publhealth-090419-102529 159-173 10.1146/annurev-publhealth-090419-102529 33035427

[2] : Critical health literacy in pandemics: the special case of COVID-19. *Health Promot Int* .2021;36(5) : 10.1093/heapro/daaa141 1473-1481 10.1093/heapro/daaa141 33351138 PMC7799085

[3] : Critical Health Literacy.2023; 10.1093/obo/9780199756797-0216 10.1093/obo/9780199756797-0216

[4] : Understanding critical health literacy: a concept analysis. *BMC Public Health* .2013;13: 10.1186/1471-2458-13-150 150 10.1186/1471-2458-13-150 23419015 PMC3583748

[5] : Critical health literacy: a review and critical analysis. *Soc Sci Med* .2011;73(1) : 10.1016/j.socscimed.2011.04.004 60-7 10.1016/j.socscimed.2011.04.004 21640456

[6] : All Aspects of Health Literacy Scale (AAHLS): developing a tool to measure functional, communicative and critical health literacy in primary healthcare settings. *Patient Educ Couns* .2013;90(2) : 10.1016/j.pec.2012.10.019 247-53 10.1016/j.pec.2012.10.019 23206659

[7] : Refining the Factor Structure of the All Aspects of Health Literacy Scale. *Am J Health Behav* .2020;44(2) : 10.5993/AJHB.44.2.1 118-128 10.5993/AJHB.44.2.1 32019646

[8] : Developing Indicators to Measure Critical Health Literacy in the Context of Norwegian Lower Secondary Schools. *Int J Environ Res Public Health* .2022;19(5) : 10.3390/ijerph19053116 10.3390/ijerph19053116 35270807 PMC8910382

# Reviewer report: Reviewer response for version 1

**DOI:** 10.5256/f1000research.24149.r84745

**Published:** 2021-06-07

**Authors:** Gerardine Doyle

**Affiliations:** 1 College of Business and Geary Institute for Public Policy, University College Dublin, Dublin, Ireland

Thank you for a very innovative research study around critical health literacy competencies and a comparative study across two countries. The educational intervention at primary school level and the podcast for parents are novel. Below are some suggestions that may help to improve your manuscript:

**Title:**

- The title of your paper could be amended to more accurately reflect your study. The use of the word ‘claims’ in a health care setting can very easily be confused with health insurance claims! This will then need to follow through your entire manuscript. Perhaps consider using the word ‘statement’ rather than ‘claims‘ so your title might be something like the following:
  The Norwegian public’s ability to assess statements about health treatments: …

**Abstract:**

- For the above reason the Background section of the Abstract needs to be rephrased.

**Research Design:**

- Surrounding the research design, the rationale for comparing Uganda with Norway is not clearly explained to the reader. Why are Uganda and Norway comparable? A justification is necessary.

**Methods and Analysis:**

- While a rationale can be understood for a comparison of adults in one country with parents and teachers in another country (all are adults), the rationale for comparing adults in one country with primary school children in another country remains a puzzle. This is so especially because the quizzes collect data on education level attained and so can be controlled for. Again this needs to be explained more clearly to the reader.
- The structure of the Methods section is difficult for the reader to follow. For example the methods section begins with explaining through Table 1 the three ‘Health Choices Key Concepts’ of ‘Claims, Comparisons and Choices’ and then moves on to the four ‘concepts’ immediately following Box 1. The use of the identical term ‘concept’ to describe both of the above is very confusing to the reader. It is not clear how the three ‘Health Choices Key Concepts’ link or map to these four ‘concepts’. Furthermore the label of ‘concepts’ does not seem to be accurate for the four ‘concepts’. Are these concepts? Perhaps ‘contentions’ is a more accurate label. This entire section needs to be redrafted.
- It is not clear from the manuscript if the quizzes went through a plain English audit process. This should be stated.
- For an English journal publication, it might be best to move the English version of the quizzes to be Additional File 1, rather than the Norwegian version.

**Conclusions:**

- The conclusion stated in the Abstract ‘This can result in poorly informed decisions, underuse of effective interventions, and overuse of ineffective or harmful interventions’ does not align with the conclusions at the end of the manuscript. Indeed it is difficult to see how these conclusions set out in the Abstract can be made from this particular study.

Two typing errors were noted:

1. Ethical Statement, second sentence
2. Page 13 should read ‘including a statement w **h**ere people were guaranteed…’

Thank you for a very interesting piece of research and I wish you every success enhancing your paper.    

Is the work clearly and accurately presented and does it cite the current literature?

Partly

If applicable, is the statistical analysis and its interpretation appropriate?

I cannot comment. A qualified statistician is required.

Are all the source data underlying the results available to ensure full reproducibility?

Yes

Is the study design appropriate and is the work technically sound?

Yes

Are the conclusions drawn adequately supported by the results?

Partly

Are sufficient details of methods and analysis provided to allow replication by others?

Yes

Reviewer Expertise:

Health Literacy, Value measurement in health care

I confirm that I have read this submission and believe that I have an appropriate level of expertise to confirm that it is of an acceptable scientific standard, however I have significant reservations, as outlined above.

# Response

**Published:** 2021-07-14

**Authors:** Astrid Dahlgren

Thank you for considering our paper and for this useful feedback. 

 We have revised the manuscript and considered all suggested revisions carefully.

 Our revisions and comments are provided below:

 1.    Suggestion to change the name “Claim”.

**Our response:** we understand that this term may be understood differently based on context. However, this term has been evaluated for relevance in several country contexts and be considered as the most precise term to be used. The Claim Evaluation Tools is also the name of the item bank this study was based on and thus cannot be changed. 

 2.    Suggestion to add rationale for comparing Uganda and Norway

**Our response:** the rationale for this comparison has now been added.

 3.    Suggestion to add rationale for comparing children and adults.

**Our response:**  the rationale for this comparison has now been added.

 4.    Suggestion to restructure the methods section describing the Key Concepts list and the claim Evaluation Tools item bank.

**Our response:**  these sections have now been revised accordingly to improve clarity.

 5.    Suggestion to clarify whether the tests went through a plain English audit process. The test was based on a set of MCQS translated from the English to Norwegian. 

**Our response:** statement now added to the manuscript. 

 6.    Suggestion to replace the Norwegian version of the tests with the English version in the additional file. 

**Our response:** F1000 publishes international studies reporting evidence from a range of contexts. The evaluation instruments used in this study was designed and adapted to a Norwegian setting. Consequently, our study describes the validation and results of the Norwegian tests. Responses and psychometric properties of the tests may be different for the English versions of these test. Thus, we believe it to be most appropriate to include the tests that was used in the study. However, examples of the questions in English has been provided in the manuscript. Other studies have used the English versions of the MCQs in other contexts (such as in Uganda).  

 7.    Suggestion to revise the conclusion taking into account: the conclusion stated in the Abstract ‘This can result in poorly informed decisions, underuse of effective interventions, and overuse of ineffective or harmful interventions’ does not align with the conclusions at the end of the manuscript. Indeed, it is difficult to see how these conclusions set out in the Abstract can be made from this particular study.

**Our response: **the sentence has been deleted.

 8.    Typos were corrected were noted

# Reviewer report: Reviewer response for version 1

**DOI:** 10.5256/f1000research.24149.r80622

**Published:** 2021-03-17

**Authors:** Julia Lühnen

**Affiliations:** 1 Clinic for Internal Medicine I, Martin Luther University Halle-Wittenberg, Halle (Saale), Germany; 2 Institute for Health and Nursing Science, Medical Faculty, al Faculty, Martin Luther University Halle-Wittenberg, Halle (Saale), Germany

The authors provided a very interesting and highly relevant paper. Critical health literacy and the ability to assess the trustworthiness of claims about healthcare are prerequisites for informed choices. For the assessment of public health literacy, only self-reported measures are used in most of the studies.

 The authors performed a cross-sectional study to assess the Norwegian public’s ability to assess treatment claims. The questionnaires, assessing understanding, are based on the key concepts people need to understand to assess healthcare claims, developed in the IHC project. The key concepts and the item bank are regularly up-dated, open access resources. The item bank included at least four multiple-choice questions for 32 Key Concepts. From this item pool four questionnaires were designed and randomly assigned to the participants.

 771 out of 4500 invited Norwegian adults responded. Rasch analysis were performed to evaluate the psychometric properties, the percentage of the Norwegian population that understands each key concept was calculated, the results were compared to the results of a previous study in Uganda and analyses were conducted to explore associations between understanding of the key concepts and demographic characteristics. The results are reported in detail. The authors’ discussed strength and limitations of their work and provided implications for practice and future research.

 I have only few comments to optimize the manuscript:

- The description of the results in the abstract and on page 7 last paragraph is confusing. Maybe there better way providing the results than using “…more than half understood x...” and “…less than half…”?
- Page 6 in the paragraph “Rasch analysis”: There is a repetition of the sentence “Rasch analysis can be used for both dichotomous and polytomous data.”
- The authors used the STROBE checklist. Looking at reporting guidelines for online surveys (e.g. CHERRIES, Eysenbach 2004), some aspect are not entirely addressed such as the number of items in each questionnaire, number of screens / pages, completion rate (besides covariates) or the handling of incomplete questionnaires.

Is the work clearly and accurately presented and does it cite the current literature?

Yes

If applicable, is the statistical analysis and its interpretation appropriate?

I cannot comment. A qualified statistician is required.

Are all the source data underlying the results available to ensure full reproducibility?

Yes

Is the study design appropriate and is the work technically sound?

Yes

Are the conclusions drawn adequately supported by the results?

Yes

Are sufficient details of methods and analysis provided to allow replication by others?

Yes

Reviewer Expertise:

evidence-based health information, informed decision-making, trainings in evidence-based medicine

I confirm that I have read this submission and believe that I have an appropriate level of expertise to confirm that it is of an acceptable scientific standard.

# Response

**Published:** 2021-07-14

**Authors:** Astrid Dahlgren

Thank you for considering our paper and for this useful feedback.

 We have revised the manuscript and considered all suggested revisions carefully.

 Our revisions and comments are provided below:

1. The description of the results in the abstract and on page 7 last paragraph is confusing. Maybe there better way providing the results than using “…more than half understood x...” and “…less than half…”?

**Our response: **these sections have now been revised accordingly to improve clarity.

1. Page 6 in the paragraph “Rasch analysis”: There is a repetition of the sentence “Rasch analysis can be used for both dichotomous and polytomous data.”

**Our response:** typo is corrected.

  

1. The authors used the STROBE checklist. Looking at reporting guidelines for online surveys (e.g. CHERRIES, Eysenbach 2004), some aspect are not entirely addressed such as the number of items in each questionnaire, number of screens / pages, completion rate (besides covariates) or the handling of incomplete questionnaires.

**Our response: **a description of the number of items per questionnaire has now been added to the description of the questionnaires in the methods section. Responses were mandatory in the software we used and thus all questionnaires were complete (thus it was not necessary to handle any incomplete questionnaires)

 For missing values on specific questions, we used multiple imputation with chained equations to account for the uncertainty introduced by missing values of the post-stratification variables sex, region of residence, and educational level. This is stated under the subheading “Survey analysis”
